# Isotretinoin and psychopathology: a review

**DOI:** 10.1186/1744-859X-8-2

**Published:** 2009-01-20

**Authors:** Vassilis P Kontaxakis, Demetris Skourides, Panayotis Ferentinos, Beata J Havaki-Kontaxaki, George N Papadimitriou

**Affiliations:** 1 Athens University Medical School, First Department of Psychiatry, Eginition Hospital, Athens, Greece

## Abstract

Isotretinoin, a synthetic oral retinoid that is used against severe nodulocystic acne, has been associated with various psychiatric side effects such as depression, suicidality and psychotic symptoms. A great number of reports on its effects have been published since its introduction into the market. However, a causal relationship has not been established and the link between isotretinoin use and psychiatric events remains controversial. The present paper reviews the available evidence regarding the association of isotretinoin and psychiatric side effects. All published material reporting psychiatric side effects following isotretinoin treatment, including case reports, case series, reports from adverse drug event reporting systems, prospective surveys and retrospective case-control studies, are presented. In addition, the neurobiology of the retinoids and possible biological mechanisms that may lead to psychopathology are described.

## Introduction

Retinoids represent a family of compounds that includes vitamin A, its derivatives and synthetic molecules that are chemically related to vitamin A. Isotretinoin (13-cis retinoic acid) is a synthetic oral retinoid that is used against severe, recalcitrant, nodulocystic acne, not responding to other therapies. It was introduced into the market as Accutane, by Hoffman-La Roche, in 1982, mainly in an attempt to improve biological activity and minimise the side effects of vitamin A compounds that were used as an effective acne treatment prior to the development of isotretinoin. It is also used for a number of other dermatological diseases such as psoriasis, ichthyasis, dermatological lesions in systemic lupus erythematosus, in the prevention of various types of skin cancer, or even as adjunctive therapy of acute promyelocytic leukemia. The side effect profile of isotretinoin includes skeletal system symptoms (arthralgia, osteoporosis), haematological (pancytopenia), ocular (corneal opacities conjunctivitis, optic neuritis, cataract) and dermatological symptoms (mild acne flare, rash, skin peeling, alopecia, photosensitivity) as well as hyperlipidemia [1-3]. Isotretinoin is also considered highly teratogenic and is classified as US Food and Drug Administration (FDA) Pregnancy category X [3]. Its use is therefore contraindicated during pregnancy. In the USA, the FDA has recently introduced the iPledge programme in an attempt to minimise the risk of pregnancy in female patients receiving isotretinoin.

Early after its release on the market, isotretinoin use was linked with psychiatric side effects such as depression, suicidal ideation and psychosis. The first report of psychiatric side effects came in 1982 by Meyskens, who had been using isotretinoin for patients with advanced cancer. He reported that 25% of his patients developed depressive symptomatology and suicidality [4]. In 1983, Hazen *et al*. reported depression in 6 of 110 patients treated for acne or a keratising disorder [5]. During the next few years, a large number of case reports, case series, reports from the Adverse Drug Event Reporting Systems (ADERS), some retrospective and prospective studies were published, linking isotretinoin with depression and suicidality (mainly) or even psychotic symptoms. In 2002, the American Academy of Dermatology invited a panel of experts to participate in a consensus conference, in order to produce an opinion regarding the safe use of isotretinoin. At the same time a second panel of experts examined the available studies on psychiatric side effects. They concluded that there were flaws in the methodology of the available studies and more scientific data was needed to draw conclusions about psychological effects. They also stated that there is not enough basic science literature about the effects of retinoids on adult brain function.

The objective of this study was to review available evidence regarding the association of isotretinoin and psychopathology.

## Literature search

We searched the MEDLINE and EMBASE databases for papers published from 1982 until March 2008 reporting psychiatric side effects following isotretinoin treatment. Keywords used were: 'isotretinoin', '13-cis retinoic acid', 'psychiatric', 'depression', 'affective', 'suicide', 'psychosis' and 'violence'. In addition, the reference sections of the identified papers and main reviews were screened.

Furthermore, several basic science papers providing evidence for potential implication of the retinoids in the etiopathogenesis of major psychiatric disorders were reviewed.

## Outcome of the literature search

A great number of single case reports, case series and reports from ADERS were found. Tables 1, 2 and 3 summarise these reports [4-28]. As shown in the tables, there are consistent ADERS reports of depression, suicide attempts, psychosis and aggression associated with isotretinoin use from many countries. The most compelling data come from the FDA in the USA where 4,992 cases of various psychiatric side effects were reported during the period from 1982 till August 2004, including 192 suicide attempts. It should be mentioned that isotretinoin ranked 4th in the top 10 of all drugs in the FDA database that were associated with a risk of depression as a side effect. In addition, psychiatric side effects are consistently higher for isotretinoin than for other acne treatments as reported by the World Health Organization in 1998 (Table 4).

**Table 1:** Case reports linking isotretinoin and psychiatric side effects

**Reference**	**Year**	**No. of patients**	**Psychiatric adverse effect**
Lindemayr [6]	1986	1	Suicide attempt
Burkett and Storrs [7]	1987	1	Depressive mood
Villalobos *et al*. [8]	1989	1	Psychosis
Hepburn [9]	1990	1	Suicide attempt
Gatti and Serri [10]	1991	1	Depression/suicide attempt
Aubin *et al*. [11]	1995	1	Suicide attempt
Cotterill and Cunliffe [12]	1997	1	Depression/suicide attempt
Cott and Wissner [13]	1999	1	Bipolar disorder
Middelkoop [14]	1998	1	Depression/suicide attempt
Ng *et al*. [15]	2001	1	Depression/suicide attempt
Poblete AC *et al*. [16]	2006	1	Panic attacks
Bachmann *et al*. [17]	2007	1	Depression/suicidal ideation

**Table 2:** Case series linking isotretinoin and psychiatric side effects

**Reference**	**Year**	**No. of patients**	**Psychiatric adverse effect**
Meyskens [4]	1982	2	Psychological changes
Hazen *et al*. [5]	1983	6	Depression
Bruno *et al*. [18]	1984	22	Depressive symptoms
Bigby and Stern [19]	1983	3	Depression/violent behaviour
Scheinman *et al*. [20]	1990	7	Depression: suicide attempt (1)
Duke and Guenther [21]	1993	2	Depression
Bravard *et al*. [22]	1993	3	Depression: suicide attempts (2)
Byrne *et al*. [23]	1998	3	Depression: suicide attempt (1)
Barak *et al*. [24]	2005	5	Affective psychosis/suicide attempts (3)

**Table 3:** Adverse drug event reports linking isotretinoin and psychiatric side effects

**Reference**	**Year**	**No. of patients**	**Psychiatric adverse effect**
US Food and Drug Administration [25]	1982 to 2004	4,992	Various psychiatric side effects: 192 suicide attempts
Canada [26]	1983 to 2003	56	Depression
UK [27]	1982 to 2006	463	Suspected psychiatric events: 25 completed suicides
Australia [28]	1985 to 1988	12	Depression: Suicide attempts (2)

**Table 4:** Number of cases of suicide, attempted suicide and suicide ideation associated with acne medications.

**Acne medication**	**Period**	**Suicide**	**Suicide attempt**	**Suicide ideation**	**Total**	**Estimated patient exposure**	**Adverse drug reactions per million**
Dianette	1980 to 1998	-	3	-	3		
Doxycycline	1965 to 1998	-	0	-	0		
Minocycline	1971 to 1998	-	2	-	2		
Oxytetracycline	1965 to 1998	-	0	-	0		
Tetracycline	1964 to 1998	-	3	-	3		
Total combined		-	8	-	8	300 million	0.03
Roaccutane	1982 to 1998	47	67	56	170	6 million	28.34

Source: World Health Organization, 1998

Furthermore, a number of retrospective and prospective studies outlined below were located.

### Retrospective studies

Jick *et al*. published a retrospective cohort study using the Saskatchewan and UK public health data bases [29]. Their study included 7,195 isotretinoin users in Saskatchewan and 340 in UK and 13,700 antibiotic users in Saskatchewan and 676 in the UK. They compared the psychiatric adverse effects between the isotretinoin and antibiotic users and also in the isotretinoin users alone pre and post treatment. The authors found no differences in the relative risk for depression and psychosis between the isotretinoin and antibiotic users. They also found no difference in the relative risk of completed suicide or attempts between the two groups in the Saskatchewan sample, whereas in the UK sample only one suicide attempt was reported (too few to draw a conclusion for relative risk). No difference was found in the incidence of psychiatric side effects for the isotretinoin users before and after treatment. It should be mentioned that this study was criticised for underestimating depression.

In a prescription analysis conducted in 2,281 patients identified in a database as having taken isotretinoin or an antidepressant it was found that patients receiving isotretinoin were no more likely to take an antidepressant [30].

The United Health Care Study found a statistically significant increase in depression in isotretinoin users when depression was defined as the coding for diagnosis and/or antidepressant medication use [31].

Mental health services utilisation was retrospectively studied in the members of the Israeli defence forces [32]. The study included 1,419 patients treated with isotretinoin and 1,102 patients with psoriasis who received other treatments. The mental health services utilisation in the isotretinoin group was 17.2% vs 12.5% in the psoriasis group, a difference that was considered statistically significant.

The first controlled study to find a statistically significant association between isotretinoin and depression was recently published [33]. This case-crossover study investigated a first diagnosis or hospitalisation for depression and antidepressant treatment prescription in 30,496 subjects who received isotretinoin therapy from 1984 to 2003. Those who received an antidepressant in the 12 months prior to the diagnosis of depression were excluded; 126 (0.4%) cases met inclusion criteria for depression. Exposure to isotretinoin in a 5-month risk period immediately prior to the diagnosis of depression was compared to a 5-month control period separated from the risk period by a 2-month 'washout' period. The number of cases exposed to isotretinoin in the 5-month risk and control periods were 41 (32.5%) and 28 (22.2%), respectively. The adjusted relative risk of isotretinoin associated with depression was 2.68.

### Prospective studies

Hull and Demkiw-Bartel [34] studied 121 patients treated with isotretinoin and found evidence of depression in 5 of them that persisted during the course of treatment.

Chia *et al*. [35] studied 132 patients with moderate to severe acne treated with isotretinoin or an antibiotic. The patients were assessed for depression before and after treatment. The authors found no differences after the treatment between the two groups.

The relationship between isotretinoin and depression was studied in a controlled cohort study [36]. Depression was assessed at baseline and after 2 months of treatment in 2 groups of patients, one receiving isotretinoin (n = 100) and the other receiving an oral (n = 41) or topical (n = 59) antibiotic (control group). No correlation between isotretinoin use and the development of depression was found.

It is worth noting that a potential source of confusion in the aforementioned clinical reports is that dermatological disease itself is considered by many authors a risk factor for depressive symptoms. Acne has been associated with depression, suicidal ideation and other psychological problems such as anxiety, embarrassment and low self-esteem [37-39]. Others have considered the psychiatric side effects as an idiosyncratic reaction to isotretinoin [40].

## Discussion

In order to establish a causal relationship between a drug and an adverse effect, a temporal relationship between drug administration and the onset of the adverse effect is necessary. Positive cases of challenge, de-challenge and re-challenge with the drug and plausibility for a biological role are also important for this association.

According to the Diagnostic and Statistical Manual of Mental Disorders version 4 text revision (DSM-IV-TR) diagnostic criteria, a diagnosis of substance-induced mood or psychotic disorder can be made if the symptoms develop during or within a month of substance intoxication or withdrawal. In addition, the symptoms must not precede the onset of the substance use and must not be substantially in excess of what would be expected given the type or amount of the substance used or the duration of use [41]. If there is a history of non-substance-induced mood or psychotic disorder, the diagnosis is doubtful. Most of the authors reporting single cases describe the onset of psychiatric side effects within 1 month of the beginning of treatment with isotretinoin. However, depression, in specific, has been reported as early as 1 day and up to 4 months after initiating isotretinoin treatment [5,7,10,12,14,15,17,18,20-23,34]. Dr Marilyn Pitts, a former safety evaluator for the FDA, reported 41 cases of positive de-challenge and re-challenge between 1982 and 1998 [42]. Of these, 28 were depressed, 5 were psychotic, 5 had an unspecified mood disorder and 3 had suicidal ideation.

Studies with animal models also indicate that exposure to isotretinoin may result in depressive-like behaviour, although the results in this area are controversial. Finally, there is biological evidence that retinoids in general can influence the central nervous system (CNS) and in particular neuronal development, neurotransmitters and systems known to be involved in the pathogenesis of psychiatric disorders.

Preliminary evidence for toxic effects of retinoids on the CNS comes from hypervitaminosis A, a toxic condition caused by the excess intake of vitamin A. Vitamin A is one of the fat soluble vitamins. It can be found in nature in various forms. In foods of animal origin the main forms are an alcohol (retinol), an aldehyde (retinal) or an acid (retinoic acid, RA). Precursors of the vitamin, called provitamins, are present in foods of plant origin and most of them belong to the carotenoids. There are over 600 carotenoids in nature and approximately 50 of them can be metabolised to Vitamin A [43]. β-Carotene is the most prevalent carotenoid in the food supply that has provitamin A activity. Retinol, the main form of vitamin A, is converted into retinyl esters in the small intestine and then further metabolised to retinol. Retinol is then inserted into chylomicrons for transport to the liver where it can be hydrolysed back to retinal when required. The utilisation of retinol in the peripheral tissues requires the irreversible conversion to retinoic acid. The majority of vitamin A effects are mediated by retinoic acid, which binds to receptors of the nuclear receptor superfamily and regulates gene expression [44]. Retinal is the essential form of vitamin A required for normal vision. Vitamin A (in the form of retinoic acid) has also an important role in the normal functioning of the immune system, especially T cell mediated immunity and natural killer activity. In the developing fetus, vitamin A is essential for normal morphogenesis, growth and cell differentiation. Hypervitaminosis A was first noted in the 16th century by Arctic explorers who ate polar bear liver or seal liver [45]. They reported symptoms of drowsiness, irritability, severe headaches, nausea and 'irrational' behaviour. In 1943, Rodahl and Moore attributed the polar bear liver toxicity to the large amounts of vitamin A ingested (13,000 IU to 18,000 IU/g: the maximum non-toxic daily intake is 10,000 IU) [46]. This hypothesis was confirmed by scientists using laboratory animals.

The reports of the European explorers of the North Pole describing emotional aberrations among the indigenous populations seem relevant. In their reports, an explosive outburst was described, which was named *Pibloktoq *by the Inuit Eskimos and referred to by other names in Siberia and elsewhere. In its classical description, *Pibloktoq *is characterised by a prodromal period of hours or days during which the person seems to be mildly irritable or withdrawn. Then, the person becomes wildly excited, starts shouting with no cause, tearing off clothes, throwing objects, mimicking screams of birds or animals and running frantically onto the tundra or ice pack placing him/herself in considerable danger. Sometimes, convulsive seizures may follow and finally stuporous sleep or coma lasting for up to 12 h. Amnesia for the experience is usually reported by the victims. Although *Pibloktoq *is a culture-bound syndrome, there are cases in which hypervitaminosis A has been hypothesised to be the underlying cause [45,47].

The entity of hypervitaminosis A is today unquestionable. It is caused by overconsumption of preformed vitamin A and not carotenoids, which are considered safe. Acute toxicity is relatively rare and is seen after administration of 150 mg in adults and 100 mg in children [43]. Symptoms include irritability, headache, nausea, vomiting, diplopia (due to increased intracranial pressure), seizures and exfoliative dermatitis [45].

Chronic vitamin A intoxication is seen in adults who ingest 15 mg/daily of vitamin A for a period of several months and in children who ingest 6 mg/daily [43]. The clinical manifestations include dry skin, glossitis, alopecia, hyperlipidemia, bone pain, increased intracranial pressure with headaches, diplopia, papilledema, irritability, fatigue, loss of energy, loss of interest, depression and sometimes psychotic symptoms [45].

The research in the effects of retinoic acid on the CNS has focused on the developing brain after the observation that isotretinoin (13-cis retinoic acid) is highly teratogenic for the CNS. Exposure of the fetus to the drug may cause a large number of birth defects, several of which involve the CNS (exencephaly, prosencephaly, hydrocephalus). More recent work, however, has suggested that retinoic acid may influence the adult brain as well [48,49]. This research is relevant to the reports of psychiatric symptoms in acne patients treated with isotretinoin.

A fundamental role of retinoic acid is the regulation of cell proliferation and differentiation via the regulation of gene transcription [50]. In the embryo this is important for the control of growth of many organs and systems, including the CNS. These functions are carried over into the adult, where the retinoic acid controls the proliferation and differentiation of the cells of the respiratory, urinary and intestinal tracts, the bones and the skin. Retinoic acid in these cells is obtained from the plasma retinol after oxidisation to retinaldehyde and then further oxidisation to retinoic acid. Retinoic acid then enters the nucleus and binds to retinoic acid receptors to activate gene transcription. Two families of receptors, retinoic acid receptors (RARs) and retinoid X receptors (RXRs), are active in retinoid-mediated gene transcription. Retinoid receptors regulate transcription by binding as dimeric complexes to specific DNA sites, the retinoic acid response elements, in target genes. The receptors can either stimulate or repress gene expression in response to their ligands. RAR binds all-trans retinoic acid and 9-cis retinoic acid, whereas RXR binds only 9-cis retinoic acid. The RXR receptors can act independently of ligand, (that is, ligand activation may not be necessary for the function of this receptor). 13-cis Retinoic acid (isotretinoin) binds weakly to the RA receptors. However, there is evidence that 13-cis retinoic acid is isomerised to all-trans retinoic acid in tissues and thus acts like all-trans retinoic acid to regulate transcription via the RA receptors [51]. RA receptors are distributed widely in the adult brain [48,49]. However, RA itself is much less widely distributed [52]. The regions of the brain that exhibit RA signalling include the limbic system, in particular the hippocampus and the medial prefrontal cortex, the cingulate cortex and subregions of the thalamus and hypothalamus [48,49].

Recent data have demonstrated that the hippocampus is one of the brain regions where new neurons are constantly born. This is a phenomenon called neurogenesis. One of the theories for the pathogenesis of depression suggests a decreased hippocampal and prefrontal cortex neurogenesis [53,54]. Antidepressant treatment seems to lead to an increase in neurogenesis, which is chronologically seen during the same period as the clinical improvement. Another irregularity in the hippocampus associated with depression is the reduction of the hippocampal volume, a finding that is correlated with prognosis (as measured by the number of hospitalisations and number of days with depression). The treatment of mice with retinoic acid results in both decreased hippocampal neurogenesis and a reduction in the hippocampal volume [55,56]. Therefore, if the effect of RA on hippocampal neurogenesis is replicated in humans, this could provide a plausible biological mechanism mediating RA's depressogenic effects.

Recent quantitative analyses have demonstrated that the concentrations of retinoic acid in the adult brain are higher in the striatum and the nucleus accumbens, in a way similar to dopamine. The enzyme retinaldehyde dehydrogenase 1 (RALDH1) which is present in the dopaminergic terminals that innervate the striatum from the ventral tegmental area is necessary for the synthesis of RA in these areas. In addition, RA seems to modulate the action of dopamine by regulating the D2 receptor [57].

Krezel *et al*. [57] reported that in adult mice, single and compound null mutations in the genes for specific retinoic acid receptors (RARβ and RXRβ and γ) resulted in locomotor defects related to dysfunction of the mesolimbic dopaminergic signalling pathway. The expression of D1 and D2 receptors was reduced in the ventral striatum of mutant mice and the response of double null mutant mice to cocaine, which affects dopamine signalling in the mesolimbic system, was blunted. The authors concluded that retinoic acid signalling defects may contribute to pathologies such as Parkinson disease and schizophrenia.

Goodman described three lines of evidence suggesting that retinoids may be implicated in the pathogenesis of schizophrenia [58]. First, several manifestations similar to those caused by retinoid dysfunction are found in patients with schizophrenia and their relatives. These manifestations include thought disorder, enlarged ventricles, agenesis of the corpus callosum and microcephaly. The second line of evidence implicating retinoids in the genetic aetiology of schizophrenia is the occurrence of known genetic markers in schizophrenia (candidate susceptibility genes), which happen to be loci of retinoid pathways or metabolic cascades (such as 6p22, 22q12-13). Finally, the transcriptional activation of dopamine D2 receptor and other schizophrenia candidate genes, such as the glutamate receptors, is regulated by retinoic acid. In a more recent work by Rioux and Arnold [59] it was reported that the expression of retinoic acid receptor α is increased twofold in the granule cells of the dentate gyrus in schizophrenia. The authors concluded that the evidence provided supports the hypothesis that retinoid pathway dysregulation may be an important factor in the aetiology of the disease.

Apart from schizophrenia, dopamine has also been implicated in depression. The dopamine hypothesis of depression supports a diminished dopaminergic neurotransmission mainly in the prefrontal cortex. Psychomotor retardation, lack of motivation, and inability to concentrate and experience pleasure are the prominent features of depression linked with reduced dopamine transmission [60]. Retinoic acid increases the expression of genes involved in dopamine signal transduction. Therefore, the direction of its effect is the opposite of what would be expected for an agent that promotes depression. It is hypothesised, however, that an initial induction of the dopaminergic system results over time in negative feedback and a long-term decline in some elements of dopaminergic transmission. It is interesting, though, that in postmortem brains of suicide victims treated with antidepressants, the D2 receptor is higher in number but 'lower' in ligand affinity. It is possible that the retinoic acid induction of the D2 receptor may result in a greater number of receptors with lower ligand affinity.

The evidence regarding the effects of retinoic acid in the serotonin pathways is controversial. In 1991, Ruiz *et al*. [61] showed that retinoic acid changes the expression of serotonin in the developing hindbrain. The application of low concentration of retinoic acid to Xenopus embryos resulted in an ectopic location of serotonergic neurons and an increase in their number. More intermediate or higher doses resulted in a decrease or complete loss of serotonergic neurons, respectively.

In 2006, O'Reilly *et al*. [62] showed that the chronic administration of 13-cis retinoic acid increases depression-related behaviour in mice, whereas Ferguson *et al*. [63], in 2007, reported that the oral treatment with 13-cis retinoic acid does not increase measures of anhedonia or depression in rats. In a more recent paper by O'Reilly *et al*. [64], 13-cis retinoic acid was found to increase 5-HT1A receptor and serotonin reuptake transporter levels in vitro; the authors concluded that this may lead to decreased serotonin availability at synapses.

Bremner *et al*. [65] used PET scans to assess the effects of isotretinoin on brain functioning. This study included 28 treatment-resistant acne patients, as defined by a failed 3-month antibiotic trial. The patients were not randomly assigned to treatment with isotretinoin or placebo. Instead they had decided with their doctors to take either a second trial of an antibiotic or isotretinoin. Each patient received a PET scan at baseline and again after 4 months of treatment with an antibiotic (n = 15) or isotretinoin (n = 13). Isotretinoin but not antibiotic treatment was associated with decreased brain metabolism in the orbitofrontal cortex, a brain area known to mediate symptoms of depression. There were no differences, however, in the severity of depressive symptoms between the two groups before and after treatment. Retinoids may lead to a decrease in orbitofrontal functioning via their effect on the hippocampus. The hippocampus modulates dopaminergic function in the medial prefrontal cortex and RA-induced deficits in hippocampal function may lead to a downstream effect on orbitofrontal function.

## Conclusion

The evidence described in this review strongly suggests a link between the use of isotretinoin and psychopathology. There is a great number of reports that support this association. Interestingly, isotretinoin is the only non-psychotropic drug in the FDA's top 10 list of drugs associated with depression. By contrast, the absence of double-blind, placebo-controlled studies, some flaws in the methodology of the current literature and some contradicting results in the studies of animal models seem to be the major reasons for the lack of an established causal link between isotretinoin use and psychiatric symptoms. However, given all the evidence, the association between isotretinoin use and psychopathology seems most likely to be justified. The multiformity of reported psychiatric adverse events (depression, suicide, psychosis) is probably associated with the multiplicity of isotretinoin's effects on various neurotransmitter systems and with the various types of vulnerability of the exposed individuals. Therefore, clinicians should be on the alert for potential psychiatric side effects following treatment with isotretinoin, especially in vulnerable populations.

## Competing interests

The authors declare that they have no competing interests.

## Authors' contributions

VPK made a substantial contribution to the conception and design of the review and has been involved in drafting and critically revising the manuscript. DS has been involved in the collection of the published material and in drafting the manuscript. PF has been involved in drafting and critically revising the manuscript. BJHK has been involved in drafting and critically revising the manuscript. GNP has been involved in critically revising the manuscript and has given final approval of the version to be published. All authors read and approved the final manuscript.
